# Supplementary material for: A novel methodology for optimal land allocation for agricultural crops using Social Spider Algorithm
Source: PeerJ. 2019 Sep 17;7:e7559. doi: 10.7717/peerj.7559 (PMC6753929; doi:10.7717/peerj.7559)
Supplement: Supplemental Information 1 [file peerj-07-7559-s001.docx]

**MATLAB R 2014b**

**Test Case 1**: All crops1-2

LandAllocation=[19990,19360,19300,19850,19560,19950,19900,19990,19770,19710];

Profit=[1693298,1896279,1777505,2033016,1848432,1816961,1867616,1801864,1989149,1893177]

TotalWaterRequirement=[288826,313341,220872,348889,264301,342496,128472,262323,270651,224768]

cftool

**Test Case 2:** Cashcrops1-2

LandAllocation =[19480,19380,19550,19860,19740,19640,19850,19230,19540,19910];

Profit =[2794228,2957229,2809634,2749084,2963316,2708188,2843588,2945043,2825620,2724805]

TotalWaterRequirement =[148243,130053,159346,150352,150200,104264,109475,155784,160413,155361]

cftool

**Test Case 3:** All crops 2-4

Profit=[3001146,3089864,3209058,3056453,3273473,3090749,3130642,3081803,3242009,2980333];

LandAllocation=[39420,37870,39360,37850,39810,38820,38160,39630,39870,39950];

TotalWaterRequirement =[515656,512916,589957,478774,559598,539956,499992,519145,586117,303497];

cftool

**Test Case 4:**Cashcrops 2-4

Profit=[5127493,5199750,5164107,5099781,5190262,5176876,5211333,5151252,5222689,5031657];

LandAllocation=[39220,39990,39520,39710,39940,39410,39970,39930,39870,39260];

TotalWaterRequirement=[330159,337070,330357,336392,329127,329079,331042,336129,335243,332264];

cftool

**Test Case 5:** Allcrops 4-10

Profit=[8489990,8429414,8775562,8353324,8764654,8621349,8358018,8635288,8632631,8485651];

LandAllocation=[98870,98740,99350,99930,98340,99710,99650,99930,99250,99960];

TotalWaterRequirement = [1271342, 1324649, 1401107, 1322762, 1398019, 1372141, 1265573, 1342021, 1373892, 1329728];

cftool

**Test Case 6:** Cashcrops 4-10

Profit=[12799613,12148477,14415305,13145674,13181461,14020586,14343288,13552033,13918658,14429256];

LandAllocation=[99180,99200,99910,99460,99330,99870,98470,99590,97760,98110];

TotalWaterRequirement=[884947,408811,833065,620327,611890,766065,758177,795324,732489,792378];

Cftool

**Test Case 7:** Allcropsabove10

LandAllocation =[199930,199440,196770,199310,196000,194580,197570,194920,198020,197810];

Profit=[17161073,16204427,16783858,16779559,16662621,16858293,16743870,16951429,16957685,17120524];

TotalWaterRequirement = [2816606, 2835758, 2644178, 2891482, 2859852, 2564068, 2686619, 2719440, 2781358, 2801587];

Cftool

**Test Case 8:** Cashcropsabove10

LandAllocation=[199820,199200,198280,199720,198110,198890,199030,199980,198220,197840];

Profit = [20619028, 24272651, 22292721, 21797271, 24014996, 23814477, 22289804, 21718483, 22040536, 23240041];

TotalWaterRequirement = [1426663, 1353925, 1349861, 1400961, 1279799, 1270661, 1455944, 1385136, 1346338, 14021198];

cftool
