# Supplementary material for: A novel methodology for optimal land allocation for agricultural crops using Social Spider Algorithm
Source: PeerJ. 2019 Sep 17;7:e7559. doi: 10.7717/peerj.7559 (PMC6753929; doi:10.7717/peerj.7559)
Supplement: Supplemental Information 2 [file peerj-07-7559-s002.docx]

using System;

using System.Collections.Generic;

using System.Linq;

using System.Text;

using System.Data.SqlClient;

using System.IO;

using System.Data;

namespace Profit_Maximization

{

class Program

{

static void Main(string[] args)

{

int i, j, k, n = 10;

int c = 0, y = 0, p = 0, cw = 0, cs = 0, cm = 0, ci = 0, ch = 0;

//,p,cw,cs,cm,ci,ch

double pjlow = 0.35, pjhigh = 0.42;

double[,] f = new double[50, 50];

double[,] m = new double[50, 50];

string[] crop = new string[10];

double[] yield = new double[10];

double[] price = new double[10];

double[] CWR = new double[10];

double[] CS = new double[10];

double[] CMF = new double[10];

double[] CI = new double[10];

double[] CHL = new double[10];

double[] TI = new double[10];

double TL = 0.0, Nf = 0.0, Nm, N = 10, OF = 0.0, area = 0.0;

double[,] TNI = new double[10, 10];

double[] weight = new double[n];

double[] objectfn = new double[n];

double[] TLA = new double[n];

Random rand = new Random();

double PF = 0.3;

double[] fun = new double[n];

Random rm = new Random();

Random alpha = new Random();

Random beta = new Random();

Random gama = new Random();

double[,] male = new double[n, n];

double[,] female = new double[n, n];

double vibci = 0.0, vibbi = 0.0, vibfi = 0.0;

SqlConnection con;

con = new SqlConnection("Data Source=DESKTOP-59VMCO2\\SQLSERVER;Initial Catalog=CropPlanning;Integrated Security=True");

con.Open();

TextWriter txl = new StreamWriter(@"C:\Users\Thilakavathi\Documents\new.xls");

txl.Write("\n");

txl.Write("Iteration");

txl.Write("\t");

txl.Write("Nf,Nm");

txl.Write("\t");

txl.Write("selected spiders");

txl.Write("\t");

txl.Write("Weight of selected spiders");

txl.Write("\t");

txl.Write("Probability");

txl.Write("\t");

txl.Write("Total Land Allocation");

txl.Write("\t");

txl.Write("Random value");

txl.Write("\t");

txl.Write("snew");

txl.Write("\t");

txl.Write("Weight of snew");

txl.Write("\t");

txl.Write("Objective value");

txl.Write("\t");

txl.Write("Weight of all spiders");

txl.Write("\t");

txl.Write("Replacing spider");

TextWriter landtxl = new StreamWriter(@"C:\Users\Thilakavathi\Documents\land.xls");

landtxl.Write("\n");

landtxl.Write("Iteration");

landtxl.Write("\t");

landtxl.Write("spider,crop");

landtxl.Write("\t");

landtxl.Write("Land Allocation");

landtxl.Write("\t");

landtxl.Write("Total Land Allocation");

landtxl.Write("\t");

landtxl.Write("Profit");

TextWriter tsw = new StreamWriter(@"C:\Users\Thilakavathi\Documents\Hello.rtf");

//TextWriter itreport = new StreamWriter(@"C:\Users\Thilakavathi\Documents\itre.rtf");

SqlCommand cmd = new SqlCommand("select crop from Crop_Values", con);

SqlDataReader dr = cmd.ExecuteReader();

while (dr.Read())

{

crop[c] = dr.GetString(0);

c++;

}

dr.Close();

SqlCommand cmd1 = new SqlCommand("select yield from Crop_Values", con);

SqlDataReader dr1 = cmd1.ExecuteReader();

while (dr1.Read())

{

yield[y] = Convert.ToDouble(dr1.GetString(0));

y++;

}

dr1.Close();

SqlCommand cmd2 = new SqlCommand("select price from Crop_Values", con);

SqlDataReader dr2 = cmd2.ExecuteReader();

while (dr2.Read())

{

price[p] = Convert.ToDouble(dr2.GetString(0));

p++;

}

dr2.Close();

SqlCommand cmd3 = new SqlCommand("select CWR from Crop_Values", con);

SqlDataReader dr3 = cmd3.ExecuteReader();

while (dr3.Read())

{

CWR[cw] = Convert.ToDouble(dr3.GetString(0));

cw++;

}

dr3.Close();

SqlCommand cmd4 = new SqlCommand("select CS from Crop_Values", con);

SqlDataReader dr4 = cmd4.ExecuteReader();

while (dr4.Read())

{

CS[cs] = Convert.ToDouble(dr4.GetString(0));

cs++;

}

dr4.Close();

SqlCommand cmd5 = new SqlCommand("select CMF from Crop_Values", con);

SqlDataReader dr5 = cmd5.ExecuteReader();

while (dr5.Read())

{

CMF[cm] = Convert.ToDouble(dr5.GetString(0));

cm++;

}

dr5.Close();

SqlCommand cmd6 = new SqlCommand("select CI from Crop_Values", con);

SqlDataReader dr6 = cmd6.ExecuteReader();

while (dr6.Read())

{

CI[ci] = Convert.ToDouble(dr6.GetString(0));

ci++;

}

dr6.Close();

SqlCommand cmd7 = new SqlCommand("select CHL from Crop_Values", con);

SqlDataReader dr7 = cmd7.ExecuteReader();

while (dr7.Read())

{

CHL[ch] = Convert.ToDouble(dr7.GetString(0));

ch++;

}

dr7.Close();

for (int i1 = 0; i1 < n; i1++)

{

TI[i1] = price[i1] * yield[i1];

Console.WriteLine("Total Income of " + crop[i1] + " is :" + TI[i1]);

tsw.WriteLine("Total Income of " + crop[i1] + " is :" + TI[i1]);

}

Console.WriteLine("Total Net Income (TNI)");

int it = 0;

do

{

Console.WriteLine("\n");

Console.WriteLine("Iteration is :" + it);

tsw.Write("\n");

tsw.WriteLine("Iteration is : " + it);

txl.Write("\n");

txl.Write(it);

txl.Write("\t");

landtxl.Write("\n");

landtxl.Write(it);

//population generation

Nf = Math.Floor((0.9 - rand.NextDouble() * 0.25) * N);

Console.WriteLine("Female Spider Population(Nf) is : " + Nf);

tsw.WriteLine("Female spider population (Nf) is : " + Nf);

Nm = N - Nf;

Console.WriteLine("Male Spider Population(Nm) is : " + Nm);

tsw.WriteLine("Male spider population(Nm) is : " + Nm);

txl.Write(Nf + "," + Nm);

txl.Write("\t");

//population initializing

//Female

for (i = 0; i < Nf; i++)

{

double profit = 0;

for (j = 0; j < n; j++)

{

f[i, j] = Math.Round(pjlow + rand.NextDouble() * (pjhigh - pjlow), 3);

//l[i, j] = f[i, j];

Console.WriteLine("Female Spider Land Allocation is :" + i + "," + j + " = " + f[i, j]);

landtxl.Write("\n");

landtxl.Write("\t");

landtxl.Write("Female Spider Land Allocation is :" + i + "," + j );

landtxl.Write("\t");

landtxl.Write(f[i, j]);

area = f[i, j];

TL = f[i, j] + TL;

OF = area * (TI[j] - CWR[j]) - area * (CS[j] + CMF[j] + CI[j] + CHL[j]);

TNI[i, j] = Math.Round(OF, 3);

profit = OF + profit;

}

objectfn[i] = Math.Round(profit, 3);

Console.WriteLine("Objective function value of Female spider " + i + " is " + objectfn[i]);

landtxl.Write("\n");

landtxl.Write("\t");

landtxl.Write("Objective function value of Female spider " + i + " is ");

landtxl.Write("\t");

landtxl.Write("\t");

landtxl.Write("\t");

landtxl.Write(objectfn[i]);

tsw.WriteLine("Objective function value of Female spider " + i + " is : " + objectfn[i]);

if (i == 0)

{

TL = f[0,0]+f[0, 1] + f[0, 2] + f[0, 3] + f[0, 4] + f[0, 5] + f[0, 6] + f[0, 7] + f[0, 8] + f[0, 9] ;

Console.WriteLine("Total Land Allocation of Female Spider 0 is : " + TL);

landtxl.Write("\n");

landtxl.Write("\t");

landtxl.Write("Total Land Allocation of Female Spider 0 is : ");

landtxl.Write("\t");

landtxl.Write("\t");

landtxl.Write(TL);

TLA[i] = Math.Round(TL, 3);

}

else if (i == 1)

{

TL = f[1,0]+f[1, 1] + f[1, 2] + f[1, 3] + f[1, 4] + f[1, 5] + f[1, 6] + f[1, 7] + f[1, 8] + f[1, 9] ;

Console.WriteLine("Total Land Allocation of Female Spider 1 is : " + TL);

landtxl.Write("\n");

landtxl.Write("\t");

landtxl.Write("Total Land Allocation of Female Spider 1 is : ");

landtxl.Write("\t");

landtxl.Write("\t");

landtxl.Write(TL);

TLA[i] = Math.Round(TL, 3);

}

else if (i == 2)

{

TL = f[2,0]+f[2, 1] + f[2, 2] + f[2, 3] + f[2, 4] + f[2, 5] + f[2, 6] + f[2, 7] + f[2, 8] + f[2, 9] ;

Console.WriteLine("Total Land Allocation of Female Spider 2 is " + TL);

landtxl.Write("\n");

landtxl.Write("\t");

landtxl.Write("Total Land Allocation of Female Spider 2 is : ");

landtxl.Write("\t");

landtxl.Write("\t");

landtxl.Write(TL);

TLA[i] = Math.Round(TL, 3);

}

else if (i == 3)

{

TL = f[3, 0] + f[3, 1] + f[3, 2] + f[3, 3] + f[3, 4] + f[3, 5] + f[3, 6] + f[3, 7] + f[3, 8] + f[3, 9] ;

Console.WriteLine("Total Land Allocation of Female Spider 3 is " + TL);

landtxl.Write("\n");

landtxl.Write("\t");

landtxl.Write("Total Land Allocation of Female Spider 3 is : ");

landtxl.Write("\t");

landtxl.Write("\t");

landtxl.Write(TL);

TLA[i] = Math.Round(TL, 3);

}

else if (i == 4)

{

TL = f[4,0] + f[4, 1] + f[4, 2] + f[4, 3] + f[4, 4] + f[4, 5] + f[4, 6] + f[4, 7] + f[4, 8] + f[4, 9] ;

Console.WriteLine("Total Land Allocation of Female Spider 4 is " + TL);

landtxl.Write("\n");

landtxl.Write("\t");

landtxl.Write("Total Land Allocation of Female Spider 4 is : ");

landtxl.Write("\t");

landtxl.Write("\t");

landtxl.Write(TL);

TLA[i] = Math.Round(TL, 3);

}

else if (i == 5)

{

TL = f[5,0] + f[5, 1] + f[5, 2] + f[5, 3] + f[5, 4] + f[5, 5] + f[5, 6] + f[5, 7] + f[5, 8] + f[5, 9];

Console.WriteLine("Total Land Allocation of Female Spider 5 is " + TL);

landtxl.Write("\n");

landtxl.Write("\t");

landtxl.Write("Total Land Allocation of Female Spider 5 is : " );

landtxl.Write("\t");

landtxl.Write("\t");

landtxl.Write(TL);

TLA[i] = Math.Round(TL, 3);

}

else if (i == 6)

{

TL = f[6,0] + f[6, 1] + f[6, 2] + f[6, 3] + f[6, 4] + f[6, 5] + f[6, 6] + f[6, 7] + f[6, 8] + f[6, 9];

Console.WriteLine("Total Land Allocation of Female Spider 6 is " + TL);

landtxl.Write("\n");

landtxl.Write("\t");

landtxl.Write("Total Land Allocation of Female Spider 6 is : ");

landtxl.Write("\t");

landtxl.Write("\t");

landtxl.Write(TL);

TLA[i] = Math.Round(TL, 3);

}

else if (i == 7)

{

TL = f[7,0] + f[7, 1] + f[7, 2] + f[7, 3] + f[7, 4] + f[7, 5] + f[7, 6] + f[7, 7] + f[7, 8] + f[7, 9];

Console.WriteLine("Total Land Allocation of Female Spider 7 is " + TL);

landtxl.Write("\n");

landtxl.Write("\t");

landtxl.Write("Total Land Allocation of Female Spider 7 is :" );

landtxl.Write("\t");

landtxl.Write("\t");

landtxl.Write(TL);

TLA[i] = Math.Round(TL, 3);

}

else if (i == 8)

{

TL = f[8,0] + f[8, 1] + f[8, 2] + f[8, 3] + f[8, 4] + f[8, 5] + f[8, 6] + f[8, 7] + f[8, 8] + f[8, 9];

Console.WriteLine("Total Land Allocation of Female Spider 8 is " + TL);

landtxl.Write("\n");

landtxl.Write("\t");

landtxl.Write("Total Land Allocation of Female Spider 8 is : " );

landtxl.Write("\t");

landtxl.Write("\t");

landtxl.Write(TL);

TLA[i] = Math.Round(TL, 3);

}

}

//Male

int g = Convert.ToInt32(Nf);

for (k = 0; k < Nm; k++)

{

double profit = 0;

for (int j1 = 0; j1 < n; j1++)

{

m[k, j1] = Math.Round(pjlow + rand.NextDouble() * (pjhigh - pjlow), 3);

//l[k + g, j1] = m[k, j1];

Console.WriteLine("Male Spider Land Allocation is : " + k + "," + j1 + " = " + m[k, j1]);

landtxl.Write("\n");

landtxl.Write("\t");

landtxl.Write("Male Spider Land Allocation is : " + k + "," + j1 + " = " );

landtxl.Write("\t");

landtxl.Write(m[k, j1]);

area = m[k, j1];

TL = m[k, j1] + TL;

OF = area * (TI[j1] - CWR[j1]) - area * (CS[j1] + CMF[j1] + CI[j1] + CHL[j1]);

TNI[g, j1] = Math.Round(OF, 3);

profit = OF + profit;

}

objectfn[g] = Math.Round(profit, 3);

Console.WriteLine("Objective function values of Male spider " + g + " is " + objectfn[g]);

landtxl.Write("\n");

landtxl.Write("\t");

landtxl.Write("Objective function values of Male spider " + g + " is " );

landtxl.Write("\t");

landtxl.Write("\t");

landtxl.Write("\t");

landtxl.Write(+objectfn[g]);

tsw.WriteLine("Objective function value of Male spider " + g + " is : " + objectfn[g]);

if (k == 0)

{

TL = m[0,0]+m[0, 1] + m[0, 2] + m[0, 3] + m[0, 4] + m[0, 5] + m[0, 6] + m[0, 7] + m[0, 8] + m[0, 9] ;

Console.WriteLine("Total Land Allocation of Male Spider 0 is " + TL);

landtxl.Write("\n");

landtxl.Write("\t");

landtxl.Write("Total Land Allocation of Male Spider 0 is ");

landtxl.Write("\t");

landtxl.Write("\t");

landtxl.Write(TL);

TLA[g] = Math.Round(TL, 3);

}

else if (k == 1)

{

TL = m[1,0]+m[1, 1] + m[1, 2] + m[1, 3] + m[1, 4] + m[1, 5] + m[1, 6] + m[1, 7] + m[1, 8] + m[1, 9];

Console.WriteLine("Total Land Allocation of Male Spider 1 is " + TL);

landtxl.Write("\n");

landtxl.Write("\t");

landtxl.Write("Total Land Allocation of Male Spider 1 is " );

landtxl.Write("\t");

landtxl.Write("\t");

landtxl.Write(TL);

TLA[g] = Math.Round(TL, 3);

}

else if (k == 2)

{

TL = m[2,0]+m[2, 1] + m[2, 2] + m[2, 3] + m[2, 4] + m[2, 5] + m[2, 6] + m[2, 7] + m[2, 8] + m[2, 9];

Console.WriteLine("Total Land Allocation of Male Spider 2 is " + TL);

landtxl.Write("\n");

landtxl.Write("\t");

landtxl.Write("Total Land Allocation of Male Spider 2 is ");

landtxl.Write("\t");

landtxl.Write("\t");

landtxl.Write(TL);

TLA[g] = Math.Round(TL, 3);

}

else if (k == 3)

{

TL = m[3,0]+m[3, 1] + m[3, 2] + m[3, 3] + m[3, 4] + m[3, 5] + m[3, 6] + m[3, 7] + m[3, 8] + m[3, 9];

Console.WriteLine("Total Land Allocation of Male Spider 3 is " + TL);

landtxl.Write("\n");

landtxl.Write("\t");

landtxl.Write("Total Land Allocation of Male Spider 3 is " );

landtxl.Write("\t");

landtxl.Write("\t");

landtxl.Write(TL);

TLA[g] = Math.Round(TL, 3);

}

else if (k == 4)

{

TL = m[4,0]+m[4, 1] + m[4, 2] + m[4, 3] + m[4, 4] + m[4, 5] + m[4, 6] + m[4, 7] + m[4, 8] + m[4, 9] ;

Console.WriteLine("Total Land Allocation of Male Spider 4 is " + TL);

landtxl.Write("\n");

landtxl.Write("\t");

landtxl.Write("Total Land Allocation of Male Spider 4 is ");

landtxl.Write("\t");

landtxl.Write("\t");

landtxl.Write(TL);

TLA[g] = Math.Round(TL, 3);

}

g++;

}

//fitness calculation

double best, worst;

best = objectfn.Max();

worst = objectfn.Min();

Console.WriteLine("Best objective function is " + best);

Console.WriteLine("Worst objective function is " + worst);

tsw.WriteLine("Best objective function is : " + best);

tsw.WriteLine("Worst objective function is : " + worst);

//Weight calculation

for (i = 0; i < n; i++)

{

double u, di;

u = objectfn[i] - worst;

di = best - worst;

weight[i] = Math.Round(u / di, 3);

Console.WriteLine("Weight of spider " + i + " is " + weight[i]);

tsw.WriteLine("Weight of spider " + i + " is : " + weight[i]);

}

for (int a = 0; a < n; a++)

{

Console.WriteLine("Total Land Allocation of spider " + a + " is " + TLA[a]);

tsw.WriteLine("Total Land Allocation of Spider " + a + " is : " + TLA[a]);

}

//Distance Calculation

double[,] dij = new double[n, n];

double[] distance = new double[90];

double[] dist = new double[100];

int d = 0, d1 = 0;

for (i = 0; i < n; i++)

{

for (j = 0; j < n; j++)

{

dij[i, j] = Math.Round(Math.Sqrt(Math.Pow(weight[i] - weight[j], 2) + Math.Pow(TLA[i] - TLA[j], 2)), 3);

dist[d1] = dij[i, j];

d1++;

if (i != j)

{

dij[i, j] = Math.Round(Math.Sqrt(Math.Pow(weight[i] - weight[j], 2) + Math.Pow(TLA[i] - TLA[j], 2)), 3);

distance[d] = dij[i, j];

////Console.WriteLine("Distance between spider " + i + " and " + j + " is :" + dij[i, j]);

tsw.WriteLine("Distance between spiders " + i + " and " + j + " is : " + dij[i, j]);

d++;

}

}

}

double mindis;

mindis = distance.Min();

//Vibration Calculation

double[,] vib = new double[n, n];

double ex, dij2;

int dd = 0;

for (i = 0; i < n; i++)

{

for (j = 0; j < n; j++)

{

dij2 = Math.Pow(dist[dd], 2);

ex = Math.Exp(-dij2);

vib[i, j] = Math.Round(weight[j] * ex, 3);

////Console.WriteLine("Vibration of " + i + " and " + j + " is " + vib[i, j]);

dd++;

}

}

//vibci calculation

//vibbi calculation

//vibfi calculation

// female co-operation calculation

double wc = 0, wb, wf, dis;

string ind;

int z = 0, z1 = 0;

ind = Convert.ToString(Array.IndexOf<double>(dist, mindis));

string[] numberArray = new string[ind.Length];

int counter = 0;

double expo, expo1, exp;

double a1, b1, g1, r1;

double wbind;

wb = weight.Max();

double exp1, exp2, exp3;

wbind = Array.IndexOf<double>(weight, wb);

int wbint = Convert.ToInt32(wbind);

for (int i1 = 0; i1 < ind.Length; i1++)

{

numberArray[i1] = ind.Substring(counter, 1);

counter++;

}

if (ind.Length == 1)

{

z = 0;

z1 = Convert.ToInt32(numberArray[0]);

}

else if (ind.Length == 2)

{

z = Convert.ToInt32(numberArray[0]);

z1 = Convert.ToInt32(numberArray[1]);

}

wc = weight[z1];

a1 = alpha.NextDouble();

b1 = beta.NextDouble();

g1 = gama.NextDouble();

r1 = rand.NextDouble();

int f1, f2;

for (f1 = 0; f1 <= Nf; f1++)

{

if (weight[z1] > weight[f1])

{

dis = Math.Sqrt(Math.Pow((TLA[f1] - TLA[z1]), 2) + Math.Pow((weight[f1] - weight[z1]), 2));

expo = Math.Pow(dis, 2);

expo1 = Math.Round(Math.Exp(-expo), 3);

exp = wc * expo1;

vibci = Math.Round(exp, 3);

////Console.WriteLine("Vibci values is " + vibci);

}

dis = Math.Sqrt(Math.Pow((TLA[f1] - TLA[wbint]), 2) + Math.Pow((weight[f1] - weight[wbint]), 2));

exp1 = Math.Pow(dis, 2);

exp2 = Math.Exp(-exp1);

exp3 = wb * exp2;

vibbi = Math.Round(exp3, 3);

////Console.WriteLine("Vibbi values is " + vibbi);

for (f2 = 0; f2 < 9; f2++)

{

double rm1 = Math.Round(rm.NextDouble(), 3);

if (rm1 < PF)

{

female[f1, f2 + 1] = Math.Round(female[f1, f2] + a1 * vibci * (wc - female[f1, f2]) + b1 * vibbi * (wb - female[f1, f2]) + g1 * (r1 - 0.5), 3);

}

else

{

female[f1, f2 + 1] = Math.Round(female[f1, f2] - a1 * vibci * (wc - female[f1, f2]) - b1 * vibbi * (wb - female[f1, f2]) + g1 * (r1 - 0.5), 3);

}

////Console.WriteLine("Female cooperative of " + f1 + " and " + f2 + " is " + female[f1, f2]);

}

}

//find the median value

double[] mweight = new double[Convert.ToInt32(Nm)];

int nuf = Convert.ToInt32(Nf);

for (int mw = 0; mw < Nm; mw++)

{

mweight[mw] = weight[nuf];

nuf++;

}

Array arr = mweight;

Array.Sort(arr);

Array.Reverse(arr);

int wmm, dm, dmw1, nfm;

double nr, drt, nrt = 0, drt1 = 0, dmw, wnfm;

nfm = arr.Length / 2;

double[] DM = new double[n];

wnfm = mweight[Convert.ToInt32(nfm)];

wmm = Array.IndexOf<double>(weight, wnfm);

Console.WriteLine("Median spider is " + wmm);

Console.WriteLine("Median spider values is " + wnfm);

List<int> mglist = new List<int>();

tsw.WriteLine("Median spider is : " + wmm);

var indexes = new List<int>();

var indes = new List<int>();

var lastIndex = 0;

if (nfm == 1)

{

dm = nfm - 1;

dmw = mweight[Convert.ToInt32(dm)];

while ((lastIndex = Array.IndexOf(weight, dmw, lastIndex)) != -1)

{

indexes.Add(lastIndex);

lastIndex++;

}

indexes.Reverse();

dmw1 = indexes[0];

//dmw1 = Array.IndexOf<double>(weight, dmw);

mglist.Add(dmw1);

DM[0] = dmw1;

tsw.WriteLine("Dominant male spider is : " + dmw1);

Console.WriteLine("Dominant male spider is" + dmw1);

Console.WriteLine("Dominant male spider value is " + dmw);

}

else if (nfm > 1)

{

for (int nfm1 = 0; nfm1 < nfm; nfm1++)

{

dmw = mweight[Convert.ToInt32(nfm1)];

//var indexes = new List<int>();

//var lastIndex = 0;

//while ((lastIndex = Array.IndexOf(weight, dmw, lastIndex)) != -1)

for(int ix=0;ix<weight.Length;ix++)

{

if (weight[ix] == dmw)

{

indes.Add(ix);

}

}

indes.Reverse();

indexes.Add(indes[0]);

dmw1 = indexes[nfm1];

//dmw1 = Array.IndexOf<double>(weight, dmw);

mglist.Add(dmw1);

DM[nfm1] = dmw1;

tsw.WriteLine("Dominant male spider is : " + dmw1);

Console.WriteLine("Dominant male spider is" + dmw1);

Console.WriteLine("Dominant male spider value is " + dmw);

}

}

//Male cooperative operator

string wfind;

int number1 = 0, number2 = 0;

wfind = Convert.ToString(Array.IndexOf<double>(dist, mindis));

string[] numberArray1 = new string[wfind.Length];

int counter1 = 0;

double expo11, expo12, exp12;

double[] mo = new double[100];

int m1 = 0, k2 = 0, i3 = 0;

int nuf1 = Convert.ToInt32(Nf);

for (int i1 = 0; i1 < wfind.Length; i1++)

{

numberArray1[i1] = wfind.Substring(counter1, 1);

counter1++;

}

if (wfind.Length == 1)

{

number1 = 0;

number2 = Convert.ToInt32(numberArray1[0]);

}

else if (wfind.Length == 2)

{

number1 = Convert.ToInt32(numberArray1[0]);

number2 = Convert.ToInt32(numberArray1[1]);

}

wf = weight[number2];

for (m1 = 0; m1 < Nm; m1++)

{

if (Convert.ToDouble(m1) < Nf && Convert.ToDouble(number2) < Nf)

{

dis = Math.Sqrt(Math.Pow((TLA[m1] - TLA[number2]), 2) + Math.Pow((weight[m1] - weight[number2]), 2));

expo11 = Math.Pow(dis, 2);

expo12 = Math.Exp(-expo11);

exp12 = wf * expo12;

vibfi = Math.Round(exp12, 3);

////Console.WriteLine("Vibfi values is " + vibfi);

////tsw.Write("\n");

////tsw.Write("Vibfi value is : " +vibfi);

}

for (k2 = 0; k2 < 9; k2++)

{

if (mweight[m1] > mweight[Convert.ToInt32(nfm)])

{

male[m1, k2 + 1] = male[m1, k2] + a1 * vibfi * (wf - male[m1, k2]) + g1 * (r1 - 0.5);

mo[i3] = Math.Round(male[m1, k2 + 1], 3);

////Console.WriteLine("Male cooperative of " + m1 + " and " + k2 + " is " + mo[i3]);

i3++;

}

else

{

for (int h = 1; h < Nm; h++)

{

double wnf = weight[nuf1 + h];

nr = male[h, k2] * wnf;

nrt = nr + nrt;

drt = wnf;

drt1 = drt + drt1;

}

male[m1, k2 + 1] = male[m1, k2] + a1 * (nrt / drt1 - male[m1, k2]);

mo[i3] = Math.Round(male[m1, k2 + 1], 3);

////Console.WriteLine("Male cooperative of " + m1 + " and " + k2 + " is " + mo[i3]);

i3++;

}

}

}

//radius calculation

double r;

double sum = 0, add = 0;

//range calculation

for (i = 1; i <= N; i++)

{

add = pjhigh - pjlow;

sum = sum + add;

}

r = sum / (2 * N);

Console.WriteLine("Range r is " + r);

tsw.WriteLine("Range value r is :" + r);

List<int> tglist = new List<int>();

List<int> eglist = new List<int>();

for (i = 0; i < Nf; i++)

{

int dom = Convert.ToInt32(mglist[0]);

double fval = dij[dom, i];

if (fval < r)

{

string index = Convert.ToString(Array.IndexOf<double>(dist, fval));

eglist.Add(i);

tglist.Add(i);

}

}

if (eglist.Count == 0)

{

Console.WriteLine("No female spider for mating ");

tsw.WriteLine("No female spider for mating ");

goto Found;

}

for (int tgi = 0; tgi < mglist.Count; tgi++)

{

tglist.Add(mglist[tgi]);

}

//influence probability calculation

double[] psi = new double[n];

double wadd, wsum = 0;

double[,] newspider = new double[10, 10];

for (int ij = 0; ij < tglist.Count; ij++)

{

int tgval = Convert.ToInt32(tglist[ij]);

wadd = weight[tgval];

wsum = wadd + wsum;

}

for (int ik1 = 0; ik1 < tglist.Count; ik1++)

{

Console.WriteLine("Spider for mating is " + tglist[ik1]);

tsw.WriteLine("Spider for mating is : " + tglist[ik1]);

txl.Write(tglist[ik1]);

txl.Write(",");

}

txl.Write("\t");

for (int selw = 0; selw < tglist.Count; selw++)

{

int sw = tglist[selw];

txl.Write(weight[sw]);

txl.Write(",");

}

txl.Write("\t");

double p_sum = 0;

List<int> tgindex = new List<int>();

List<double> tgvalue = new List<double>();

for (int ik = 0; ik < tglist.Count; ik++)

{

int tgval = Convert.ToInt32(tglist[ik]);

psi[ik] = Math.Round(weight[tgval] / wsum, 3);

tgindex.Add(tglist[ik]);

tgvalue.Add(psi[ik]);

p_sum += psi[ik];

Console.WriteLine("Probability is " + psi[ik]);

txl.Write(psi[ik]);

txl.Write(",");

tsw.WriteLine("Probability is : " + psi[ik]);

}

tsw.WriteLine("Sum of probability is : " + p_sum);

Console.WriteLine("Sum of probability is :" + p_sum);

int[] sortarrin = new int[tgvalue.Count];

double[] sortarrval = new double[tgvalue.Count];

sortarrin = tgindex.ToArray();

sortarrval = tgvalue.ToArray();

Array.Sort(sortarrval, sortarrin);

foreach (var str in sortarrin)

{

Console.WriteLine("sorted array index is " + str.ToString());

}

foreach (var str in sortarrval)

{

Console.WriteLine("sorted array value is " + str.ToString());

}

txl.Write("\t");

for (int ijk = 0; ijk < n; ijk++)

{

txl.Write(TLA[ijk]);

txl.Write(",");

}

//landtxl.Write("\n");

//int il, ils;

//for (il = 0; il < n; il++)

//{

// landtxl.Write("\n");

// landtxl.Write(il);

// landtxl.Write("\t");

// for (ils = 0; ils < n; ils++)

// {

// landtxl.Write(l[il,ils]);

// landtxl.Write(",");

// }

// landtxl.Write("\t");

// landtxl.Write(TLA[il]);

//}

//Mating operation

int snew = 0;

Random rd1 = new Random();

double rdval = Math.Round(rd1.NextDouble(), 3);

Console.WriteLine("Random value is :" + rdval);

tsw.WriteLine("Random value is : " + rdval);

txl.Write("\t");

txl.Write(rdval);

double a3 = 0, b3 = 0, c3 = 0, d3 = 0;

a3 = sortarrval[0];

b3 = sortarrval[0] + sortarrval[1];

if (sortarrval.Length == 3)

{

c3 = sortarrval[0] + sortarrval[1] + sortarrval[2];

}

if (sortarrval.Length == 4)

{

c3 = sortarrval[0] + sortarrval[1] + sortarrval[2];

d3 = sortarrval[0] + sortarrval[1] + sortarrval[2] + sortarrval[3];

}

if (rdval < a3)

{

snew = sortarrin[0];

Console.WriteLine("Snew is : " + snew);

}

else if (rdval > a3 && rdval < b3)

{

snew = sortarrin[1];

Console.WriteLine("Snew is : " + snew);

}

else if (rdval > b3 && rdval < c3)

{

snew = sortarrin[2];

Console.WriteLine("Snew is : " + snew);

}

else if (rdval > c3 && rdval < d3)

{

snew = sortarrin[3];

Console.WriteLine("Snew is : " + snew);

}

int respider=0;

//for (int isn = 0; isn < n; isn++)

//{

double min = weight.Min();

int win = Array.IndexOf<double>(weight, min);

if (weight[snew] > min)

{

weight[win] = weight[snew];

objectfn[win] = objectfn[snew];

respider = win;

Console.WriteLine("Replacing spider is : " + win);

tsw.WriteLine("Replacing spider is : " + win);

}

else

{

weight[snew] = weight[snew];

}

//}

txl.Write("\t");

txl.Write(snew);

txl.Write("\t");

txl.Write(weight[snew]);

txl.Write("\t");

txl.Write(objectfn[snew]);

txl.Write("\t");

for (int ij1 = 0; ij1 < weight.Length; ij1++)

{

txl.Write(weight[ij1]);

txl.Write(",");

}

txl.Write("\t");

txl.Write(respider);

tsw.WriteLine("New spider is : " + snew);

tsw.WriteLine("Weight of new spider is : " + weight[snew]);

tsw.WriteLine("Objective function of new spider is : " + objectfn[snew]);

Console.WriteLine("Weight of new spider is :" + weight[snew]);

Console.WriteLine("Objective function of new spider is :" + objectfn[snew]);

Found:

it++;

} while (it < 500);

txl.Close();

landtxl.Close();

tsw.Close();

Console.Read();

}

}

}
